# The TolC Protein of *Legionella pneumophila* Plays a Major Role in Multi-Drug Resistance and the Early Steps of Host Invasion

**DOI:** 10.1371/journal.pone.0007732

**Published:** 2009-11-04

**Authors:** Mourad Ferhat, Danièle Atlan, Anne Vianney, Jean-Claude Lazzaroni, Patricia Doublet, Christophe Gilbert

**Affiliations:** 1 Université de Lyon, Lyon, France; 2 Université Lyon 1, Lyon, France; 3 INSA de Lyon, Villeurbanne, France; 4 Bayer CropScience, Lyon, France; 5 CNRS, UMR5240, Unité Microbiologie, Adaptation et Pathogénie, Villeurbanne, France; Charité-Universitätsmedizin Berlin, Germany

## Abstract

Pneumonia associated with Iegionnaires's disease is initiated in humans after inhalation of contaminated aerosols. In the environment, *Legionella pneumophila* is thought to survive and multiply as an intracellular parasite within free-living amoeba. In the genome of *L. pneumophila* Lens, we identified a unique gene, *tolC*, encoding a protein that is highly homologous to the outer membrane protein TolC of *Escherichia coli*. Deletion of *tolC* by allelic exchange in *L. pneumophila* caused increased sensitivity to various drugs. The complementation of the *tolC* mutation in trans restored drug resistance, indicating that TolC is involved in multi-drug efflux machinery. In addition, deletion of *tolC* caused a significant attenuation of virulence towards both amoebae and macrophages. Thus, the TolC protein appears to play a crucial role in virulence which could be mediated by its involvement in efflux pump mechanisms. These findings will be helpful in unraveling the pathogenic mechanisms of *L. pneumophila* as well as in developing new therapeutic agents affecting the efflux of toxic compounds.

## Introduction

*Legionella pneumophila* (*L. pneumophila*), the main causative agent of the Legionnaire's disease in humans is commonly found in freshwater environments where it can replicate within protozoa [1]. Development of air-conditioning systems, cooling towers and other water aerosols has created conditions for the direct access of this opportunistic bacterium to human lungs, where it can multiply within alveolar macrophages [2]. During the intracellular infectious cycle in amoeba and macrophages, *L. pneumophila* evades the phagolysosome fusion and establishes a replicative vacuole studded with ribosomes [1], [3], [4]. The subsequent modified vacuole offers a nutrient rich environment, which allows the bacteria to replicate. When nutrients become scarce, the bacteria undergo a phenotypic switch leading to the expression of virulent traits including flagella expression and an increased resistance to antibiotics and stress. Finally, *Legionellae* lyse the vacuole and the membrane host, probably by secreting pore-forming toxins [5].

Up to date, prevention of legionellosis outbreaks has been based on the control of the legionellae population in cooling towers by using chemical treatments. Little is known about the resistance of *L. pneumophila* to these biocides. One universal mechanism underlying drug resistance to various toxic compounds, namely multi-drug resistance (MDR; [6]), is the expression of efflux pumps that drive drugs outside the target cell. Five families of efflux pumps have been described on the basis of the inner membrane protein structure: the major facilitator (MF) superfamily, the ATP-binding cassette (ABC) family, the resistance-nodulation-division (RND) family, the small multi-drug resistance (SMR) family and the multi-drug and toxic compound extrusion (MATE) family [7], [8]. The ABC-efflux pump system is ATP-dependent whereas MF, RND and SMR systems use the proton motive force. MATE-family transporters are mainly Na+/substrate antiporters, but few members have been described as H+/drug antiporters.

SMR and MATE systems have been described to transport drugs from the cytoplasm to the periplasm, then releasing them to the extracellular medium via porins of the outer membrane. Tripartite systems (MFS, ABC and RND) have also been described, such as the AcrA-like/AcrB-like/TolC efflux pump belonging to the RND family [9], [10]. TolC is a protein found in all Gram-negative bacteria, forming a channel through the outer membrane and interacting with the AcrA periplasmic protein. This AcrA lipoprotein anchored to the inner membrane via a lipid motif also interacts with AcrB, an integral inner membrane translocase acting as a proton/drug antiporter [11].

Besides its role in the efflux of various molecules, TolC has been recently reported to play a key role in bacterial virulence in the Gram negative bacteria such as *Francisella tularensis* [12], *Brucella suis* [13], *Salmonella enterica* serovar Typhimurium [14] and *Salmonella enteritidis* [15]. Here we report that *L. pneumophila* Lens encodes a protein homologous to *Escherichia coli* TolC. We demonstrate that the TolC protein is involved in multidrug-resistance of *L.pneumophila* and that it plays an essential role in virulence.

## Results

### *L.pneumophila* Encodes a TolC Ortholog

Using BLAST and PSI-BLAST analysis of the *L. pneumophila* Lens genome (http://genolist.pasteur.fr/LegioList/) [16], 116 ORFs were identified by significant homology with known proteins involved in efflux pump machineries of many bacteria species: 12 outer membrane factors (OMF), 16 periplasmic membrane fusion proteins (MFP) and 88 inner membrane proteins (IMP) belonging to four classes of efflux pumps (MF, ABC, RND and SMR) [8]. No member of the multi-drug and toxic compound extrusion (MATE) family was identified. Among the 12 OMF proteins identified, one shares a significant homology (36% identity and 55% similarity) with the *Escherichia coli* TolC protein and will be referred to here as *L. pneumophila* TolC.

*L. pneumophila* TolC contains a predicted leader sequence with the cleavage site located between residues 20 and 21 (VFA↓TD) and,, as a consequence is predicted to be localized in the outer membrane. TolC contains duplicate pfam02321 domains forming trimeric channels that allow export of a variety of substrates in Gram-negative bacteria [17]. The trimeric channel is composed of a 12-stranded beta sheet barrel that spans the outer membrane, and a long helical barrel that spans the periplasm. These domains are part of the TolC family domain (COG1538; [17]).

### Inactivation of the tolC Gene Has No Effect on Growth of L. pneumophila Lens

In order to identify the role of the TolC protein in *L. pneumophila*, a *tolC* mutant was constructed as described in Materials and Methods. This mutant, called MF201, is partially deleted for *tolC* by a unique insertion of a kanamycine cassette (*tolC*::*kan*), verified by PCR and Southern hybridization (data not shown).

A pUC18 derivative vector, namely pML005, was constructed by site-directed mutagenesis to obtain a plasmid that displays a previously described mutation which enhances its stability in *L. pneumophila* [18] and allows complementation experiments in derivative strains. A preliminary bioluminescence assay indeed confirmed the stability of a *lux* cassette expressing pML005 during 30 generations without selection pressure (data not shown). The strain MF201 was transformed by plasmid pML005-*tolC*, which efficiently expressed the *L. pneumophila* Lens *tolC* gene, to obtain the MF213 strain.

Because the tolC mutation has been shown to affect bacterial cell division in E. coli [19], the growth kinetics of the tolC mutant (MF201) was compared to that of the parental (Lp01) and complemented strains (MF213). MF201, MF213 and MF214 exhibited no growth defects either in liquid media (Figure S1) or on solid media (data not shown). Thus, L. pneumophila behaved as Francisella tularensis for which the deletion of tolC had no effect on bacterial growth [12].

### TolC Is Involved in MDR of *L. pneumophila* Lens

The sensitivity of strain MF201 compared to the wild type was tested towards 17 toxic compounds. The drugs were chosen from a wide range of toxic compounds which have been identified to be expelled by efflux [20]: detergents (SDS, CTAB), antibiotics (erythromycin, novobiocin, nalidixic acid, norfloxacin and tetracyclin), dyes (methylene blue, acridine orange and rhodamine), quaternary ammonium or benzalkonium chloride, intercalating agents (ethidium bromide), bile salts (sodium deoxycholate) and heavy metals (nickel, zinc and manganese). Interestingly, strain MF201 (*tolC*::*kan*) showed an increased sensitivity towards 11 compounds over the 17 tested, with a reduced MIC ranging from 2 to 16-fold (erythromycin) compared to that of the wild-type strain Lp01 (Table 1). No difference in MIC was observed in the presence of cobalt, tetracycline, nalidixic acid, acridine orange, zinc and manganese (data not shown). These results ruled out the possibility of a bacterial envelope defect leading to an non-specific release of all tested drugs, but favored an alteration of the drug efflux mechanism of the *tolC* mutant. As expected, the complemented strain MF213 displayed similar drug sensitivity compared to the parental strain. Taking together, these results support that MF201 phenotype was the result of *tolC* inactivation.

**Table 1:** Drugs susceptibility of *L. pneumophila* Lens grown on BCYE Agar.

		MIC 100 (µg/mL)^b^										
Strain^a^	Genotype	SDS	CTAB	ERY	BENZ	NOV	DEO	NOR	ETB	MB	R6G	Ni
Lp01	WT	0,125	50	0,5	100	6,25	100	12	100	100	50	800
**MF201**	***tolC*** **::** ***kan***	**0,031**	**25**	**0,031**	**25**	**3,125**	**12,5**	**6**	**25**	**50**	**25**	**400**
MF213	*tolC*::*kan*/pML005-*tolC*	0,125	50	0,5	100	6,25	100	12	100	100	50	800
**MF214**	***tolC*** **::** ***kan*** **/pML005**	**0,031**	**25**	**0,031**	**25**	**3,125**	**12,5**	**6**	**25**	**50**	**25**	**400**
Lpl701	*dotA:: kan*	0,125	50	0,5	100	6,25	100	12	100	100	50	800

a Strains used are described in Materials and Methods.

b MIC 100 were determined as the minimal inhibitory concentration leading to a complete inhibition of bacterial growth using agar dilution method (see Materials and Methods). Results were reproduced three times.

*Abbreviations:* SDS: Sodium dodecyl sulfate, CTAB: hexadecyltrimethylammonium bromide, ERY: Erythromycin, BENZ: Benzalkonium chloride, NOV: Novobiocin, DEO: Sodium Deoxycholate, NOR: Norfloxacin, ETB: Ethidium bromide, MB: Methylene Blue, R6G: Rhodamine 6G, Ni: nickel sulfate.WT: wild type.

**Figure 1:**
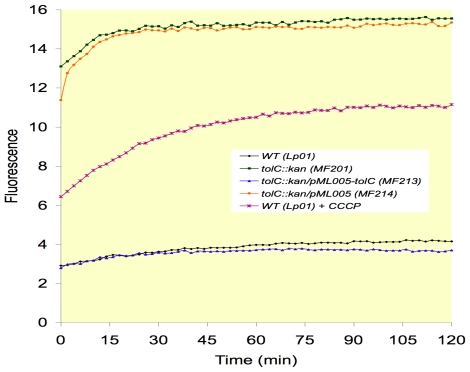
Ethidium bromide accumulation in *L. pneumophila* Lens derivatives. Bacteria were grown in liquid medium (BYE medium) at 30°C until stationary phase. The accumulation assay was done as described in Materials and Methods in presence of 0.5 mg/l of ethidium bromide. When indicated, carbonyl cyanide m-chlorophenylhydrazone (CCCP) was added (2.5 mg/l).

It is commonly assumed that ethidium bromide is a substrate of TolC-dependent efflux pumps [13]. Therefore, the accumulation of ethidium bromide in bacteria was measured in *L. pneumophila* Lens and derivatives (Figure 1). A stable level of ethidium bromide (close to 4 units of fluorescence) was observed in *L. pneumophila Lens* over a period of 120 min. The addition of carbonyl cyanide m-chlorophenylhydrazone (CCCP; 2.5 mg/L), known to disrupt the proton motive force, resulted in a rapid increase of ethidium bromide concentration inside bacterial cells. High accumulation of ethidium bromide was observed in the TolC defective mutant (MF201), which was fully reversed by the *tolC* complementation (strain MF213) (Fig. 1). This accumulation was even higher than that observed in the wild type in presence of CCCP. However, the concentration of CCCP used was low (2.5 mg) in order to limit cellular death [21] and might result in partial inhibition of efflux pump systems, which may explain the mild level of inhibition.

All these results confirm that TolC of *L. pneumophila* Lens is a component part of a functional efflux pump with wide substrate specificity and driven by the proton motive force.

### TolC Contributes to Stress Resistance in *L. pneumophila* Lens

**Figure 2:**
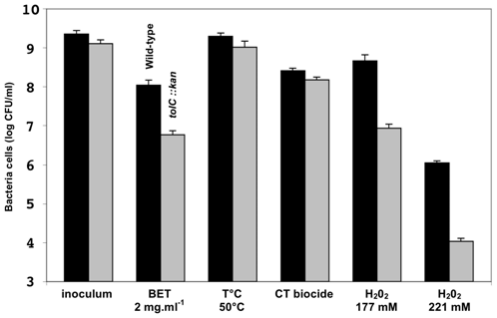
Cultivable surviving bacteria after exposition to different types of stress. Strains Lens (WT; back bars) and MF201 (*tolC::kan*; grey bars) were subjected to oxidative stress (H_2_0_2_), to thermal stress, and to the effect of a cooling tower biocide (CT) for 1 hour. Bacteria were then washed and diluted for colony enumeration on BCYE agar.

*Legionellae* were exposed to chemical compounds (ethidium bromide, H_2_O_2_, cooling tower biocides) or high temperature (50°C) for 1 hour in liquid medium. After ethidium bromide stress, the viability of MF201 was 18-fold lower (p-value = 0.0014) than that of the parental strain (Fig. 2). The sensitivity of MF201 increased as a function of the hydrogen peroxide concentration (53- (p-value = 0.003) and 102-fold (p-value = 9×10^−6^) decrease with 177 and 221 mM H_2_0_2_, respectively). In contrast, strain MF201 displayed similar resistance as the parental strain towards thermal stress and biocides used as disinfectants in cooling towers. Therefore, TolC contributes to oxidative stress resistance. Besides, these results also confirmed that the deficiency in TolC protein is not correlated with a bacterial envelope alteration inducing a general sensitivity of bacterial cells.

### The *tolC* Gene Is Essential for Virulence of *Legionella* towards Protozoa and Macrophages

**Figure 3:**
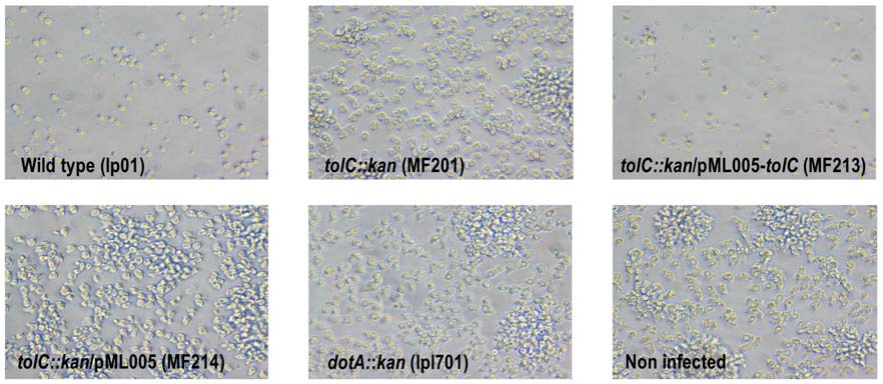
Infected monolayers of *D. discoideum* at 72 hours post-infection (X20). Monolayers were observed with a Nikon inverted microscope coupled with an Olympus camera (DP120).

The microscopic observations infections conducted in *Acanthamoeba castellanii* and *Dictyosteliun discoideum* showed that strain MF201 failed to “stress” the protozoan cells, which remained adherent even after 72 hours post-infection (Fig. 3). At this time, the wild type strain was able to lyse almost all amoebae present in the monolayer (the remaining amoebae were round) and many highly motile bacteria (virulent phenotype) were present in the media. Interestingly, the defect in virulence observed with strain MF201 was similar to that observed with strain lpl701 (avirulent control strain) known to be defective for virulence secretion factors [5]. The restoration of the virulent phenotype after complementation (strain MF213) and the fact that the empty plasmid could not restore this phenotype ruled out the possibility that the defective virulence observed with strain MF201 was due to spontaneous or secondary mutations.

**Figure 4:**
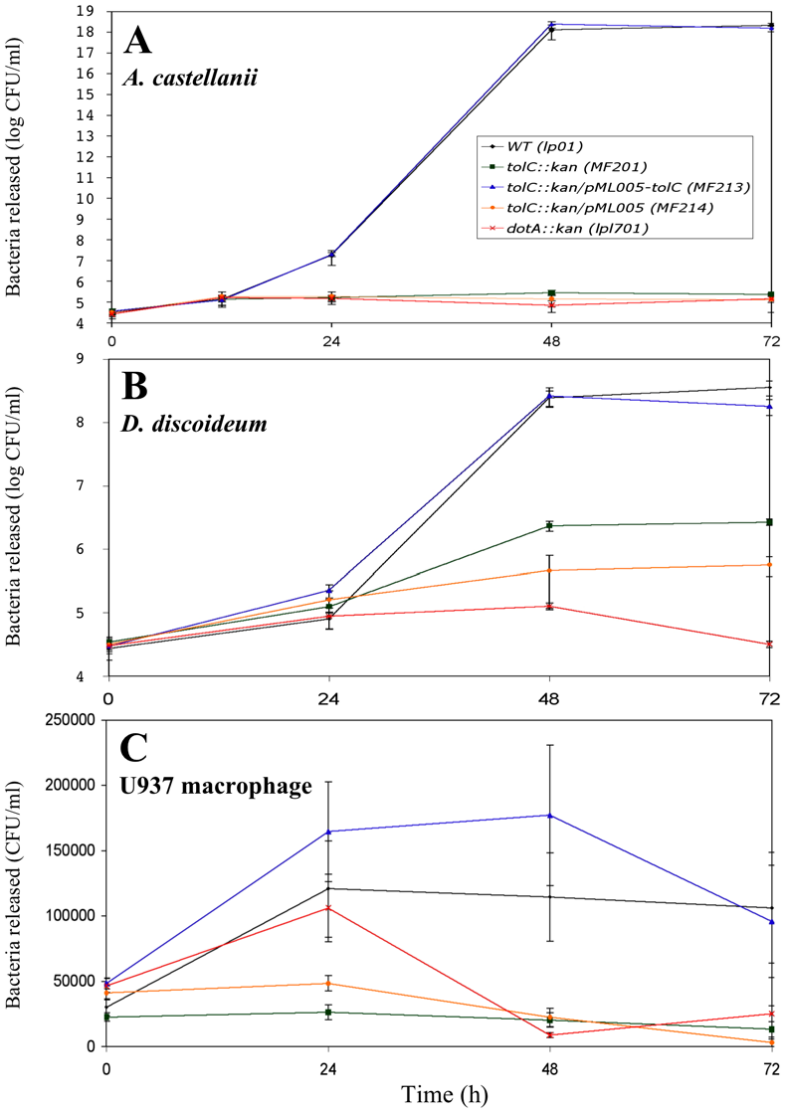
*Legionella* cells released from protozoa and macrophages. *A. castellanii* (A), *D. discoideum* (B) and U937 human monocyte-derived macrophages (C) were infected at an MOI of 10 for 1 h with *L. pneumophila* strains Lp01 (WT), MF201 (*tolC::kan*), MF213 (*tolC::kan*/pML005-*tolC*), MF214 (*tolC::kan*/pML005) and lpl701 (*dotA::kan*). At different times post-infection, the bacteria released in the supernatants were diluted in the appropriate medium (see material and methods) and spread on BCYE agar plates for colony enumeration. The initial time points (t = 0) represent the number of extracellular bacteria after 1 hour of infection. The data are representative of two independent experiments performed in triplicate and error bars represent standard deviations.

The amoebae *A. castellanii* and *D. discoideum* as well as U937 macrophages were infected with the parental strain Lp01, the TolC defective strain MF201, the complemented strain MF213, the corresponding plasmid-control strain MF214, or the avirulent *dotA* mutant. Extracellular *Legionellae* were counted at 24, 48 and 72 h (Fig. 4). As expected, infections of the three eukaryotic cell monolayers with the parental strain Lp01 showed an increase of extracellular *Legionellae* between 0 and 24 h post-infection (580-, 2.9- and 4-fold increase, respectively). Between 24 and 48 hours after infection, there was a significant increase of bacterial egress from the amoeba *A. castellanii* and to a lesser extent from *D. discoideum* (10 and 3 log increase, respectively). This burst in bacterial egress can be correlated with the switch to a virulent phenotype after multiplication within a eukaryotic host. It must be underlined that beyond 48 h of post-infection the level of bacterial egress from amoebae and macrophages remains stable.

Compared to the parental strain, there was a severe defect of egress of MF201 cells at 24 h (400-fold less bacteria from *A. castellanii* (p-value = 0.015) and 4-fold difference from U937 (p-value = 9×10^−4^)) and at 48 h (12 log less bacteria from *A. castellanii* (p-value = 0.015), 2 log less bacteria from *D. discoideum* (p-value = 2.8×10^−4^) and 5.6 fold less bacteria from U937 (p-value = 3×10^−4^)). This defect of MF201 cell egress was similar to the one observed with the avirulent *dotA* strain at 72 h (p-value>0.05) in *A. castellanii* and in U937 cells. The *tolC* complementation restored the *Legionellae* egress from the three tested eukaryotic cells (Fig. 4) and no significant difference was observed between the toxicity of parental strain (Lp01) and complemented strain MF213 (p-value>0.05) after 72 h of *A. castellanii* or U937 infections.

**Figure 5:**
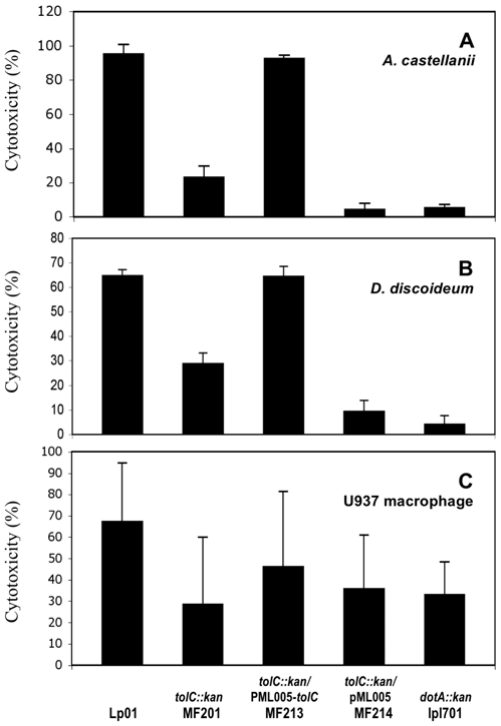
Cytotoxity of *L. pneumophila* Lens derivatives towards different hosts. *A. castellanii* (A), *D. discoideum* (B) and U937 human monocyte-derived macrophages (C) cells were infected at an MOI of 10 for 1 h with *L. pneumophila* strains: Lp01 (WT), MF201 (*tolC::kan*), MF213 (*tolC::kan*/pML005-*tolC*), MF214 (*tolC::kan*/pML005) and lpl701 (*dotA::kan*). After 48 h (*D. discoideum*) or 72 h (*A. castellanii* and U937) of infection, the monolayers were washed and the reduction of the Alamar blue dye was measured and compared to non-infected cells (100%). These data are representative of two independent experiments done in triplicate (error bars represents standard deviations).

In addition, Alamar blue dye was used to quantify the viability of eukaryotic cells present in the infected monolayers. The cytotoxicity of the parental strain towards *A. castellanii*, *D. discoideum* and U937 macrophages was respectively estimated at 95%, 64% and 67%, (Fig. 5). Compared to the wild type strain, the cytotoxicity of the *tolC* mutant was significantly lower: 23% (p-value = 1.2×10^−6^), 29% (p-value = 1.2×10^−7^) or 28% (p-value = 0.017) towards *A. castellanii, D. discoideum* or U937 macrophages, respectively. As expected, the cytotoxicity level was fully restored in the complemented strain MF213 (p-value>0.05) in the three hosts.

Taken together, these results confirm a severe defect of virulence and cytotoxicity correlated with the absence of the TolC protein in *L. pneumophila*.

### TolC Is Required for Multiplication of *L. pneumophila* at the Onset of Early Steps of the Intracellular Infectious Cycle

**Figure 6:**
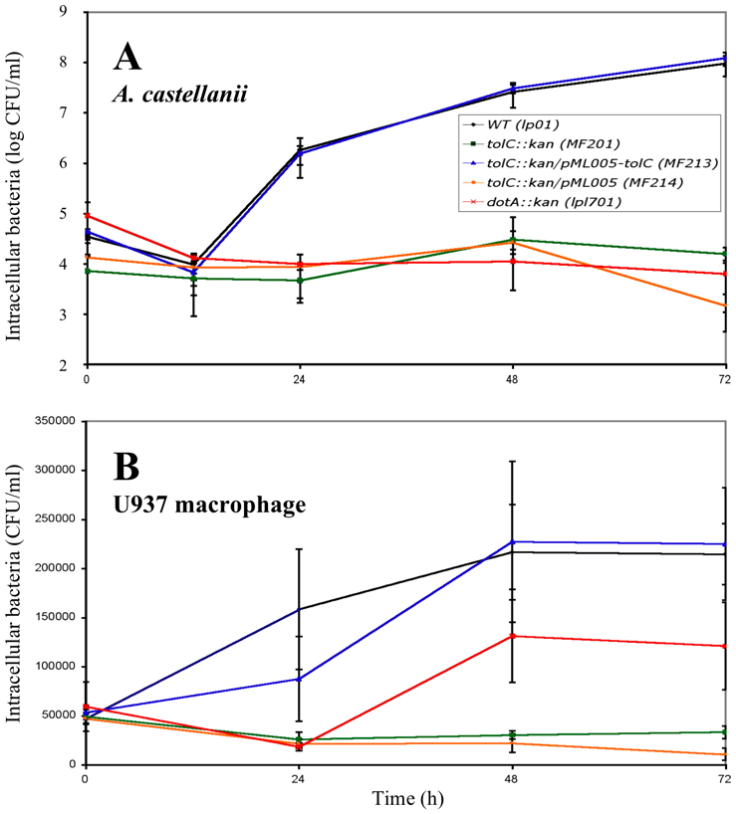
Intracellular growth of *L. pneumophila* Lens derivatives. Intracellular growth kinetics of strains Lp01 (WT), MF201 (*tolC::kan*), MF213 (*tolC::kan*/pML005-*tolC*), MF214 (*tolC::kan*/pML005) and lpl701 (*dotA::kan*) in *A. castellanii* (A) and U937 human monocyte-derived macrophages (B). These cells were infected at an MOI of 10 for 1 h with *L. pneumophila*. At different times postinfection, *A. catellanii* or U937 monolayers were lysed either hypotonically (sterile water for U937 cells) or with a mild detergent (0,04% Triton X-100 for *A. castellanii*). Aliquots were diluted immediately and plated on BCYE agar plates for enumeration of intracellular bacteria. Each experiment was performed twice in triplicate. Error bars represent standard deviations.

Intracellular *Legionellae* were followed during 72 h of infection to differentiate between two possible consequences of the TolC deficiency: a defect in intracellular multiplication or in the capacity to lyse eukaryotic cells. The intracellular concentration of the *L. pneumophila* Lens cells within *A. castellanii* was increased 52- and 14-fold during the first 24 h and the 24–48 h period, respectively (Fig. 6A). In contrast, the level of MF201 cells did not significantly increase over the 72 h of post-infection within *A. castellanii* and U937 macrophages (Fig. 6). Therefore, at 72 h post-infection, the number of MF201 intracellular bacteria was 6000 fold lower (p-value = 2.5×10^−3^) in *A. castellanii* and 6.5 fold lower (p-value = 5.2×10^−5^) in U937 macrophages compared to the parental strain Lp01. Similar results were observed using *D. discoideum* as host (data not shown). This low level of intracellular MF201 *Legionellae* could not be explained by a defect in host adherence because no difference was observed between the numbers of non-adherent parental or mutant cells (data not shown).

**Figure 7:**
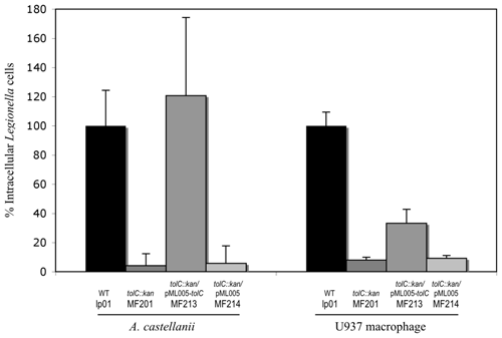
Invasion ability of *L. pneumophila* Lens derivatives strains. Host cells were infected at an MOI of 10 with strains Lp01 (WT), MF201 (*tolC::kan*), MF213 (*tolC::kan*/pML005-*tolC*), MF214 (*tolC::kan*/pML005) and lpl701 (*dotA::kan*). After 60 minutes of contact with *L. pneumophila*, the monolayers were treated 1 hour with gentamycin to kill adherent bacteria and disrupted with either distilled water (for U937 cells) or Triton 0,04% (for *A. castellanii*). Intracellular bacteria were diluted and plated on BCYE agar plates for colony enumeration. Results are expressed as the relative value (%) compared to control invasion experiment with wild-type strain Lp01. These data are representative of two independent experiments done in triplicate for which error bars represents standard deviations.

Thus, our results reveal that *Legionellae* could not efficiently initiate an infectious cycle without the TolC protein. This statement was confirmed by the results obtained after a treatment of extracellular *Legionellae* with gentamicin at the onset of host infection: the intracellular level of the mutant MF201 corresponded to less than 10% of the parental strain amount within *A. castellanii* (p-value = 3.1×10^−6^) or U937 macrophages (p-value = 0.039) (Fig. 7). This result points out the major role of TolC in the early steps of eukaryotic cell infection. However, the reference level of bacteria (Lp01) was fully recovered by the complemented strain MF213 in *A. castellanii* infection (p-value>0.05), but only partially recovered during macrophage invasion. In that case, the number of *Legionella* MF213 cells is still significantly different from the control level (Lp01) (p-value = 0.019). This latter result might be due to the genetic system we used: *tolC* gene expression in trans was under the control of a constitutive promoter, therefore expression may have been at a lower level than the parental *tolC* gene, whose expression may be fully induced by host stress signals at some steps of the infectious cycle. As a consequence, a lower level of TolC in the complemented strain compared to the wild type during macrophage infection cannot be ruled out.

## Discussion

Our analysis of the *L. pneumophila* Lens genome revealed a unique ORF sharing significant homology with the TolC protein from *Escherichia coli*, which is the prototypical outer membrane channel component involved in MDR and type I secretion [22], [23], [24]. The *L. pneumophila Lens tolC::kan* mutant was sensitive to a variety of compounds including antibiotics and detergents, which supports the involvement of the TolC protein in a functional MDR machinery with wide substrate specificity. The TolC pump activity was clearly demonstrated by the increase of ethidium bromide accumulation in the TolC deficient strain compared to the parental strain. The trans-complementation of *tolC* restored the pumping efficiency suggesting that the drug sensitivities were specifically due to the *tolC* knockout. Thus, our work confirms the role of *L. pneumophila* TolC in drug efflux mechanisms.

Moreover, the *tolC* mutant of *L. pneumophila* was clearly more sensitive to H_2_O_2_ than the wild type, which shows that TolC protects *L. pneumophila* against oxidative stress. This result is in agreement with the newly identified role of TolC protein in *S. meliloti and S. enterica* [25], [26]. Actually, oxidative stress occurs when organisms encounter reactive oxygen species (ROS) such as superoxide anion, hydrogen peroxide and hydroxyl radical. ROS are known to be produced during the oxidative burst of professional phagocytes to kill intracellular bacteria during an infection [27], [28]. Therefore, in phagocytic cells, bacteria have to cop with a stressful and hostile environment (ROS, antimicrobial peptides, degradative enzyme for example) in which multi-drug and stress resistance mechanisms can confer a selective advantage for intracellular survival in the host. The exact role of TolC in response to oxidative stress is still unclear, but it could participate in the efflux of toxic ROS in addition to ROS degradation by the bacterial periplasmic and cytoplasmic catalases and dismutases [29], [30], [31]. Moreover, in *E. coli*, *tolC* belongs to the *marA/soxS/rob* regulon including over 40 genes that promote resistance to multiple antibiotics and to superoxides [32]. Four *tolC* promoters have been described in *E. coli*, suggesting the involvement of multiple transcriptional regulatory elements in response to different environments [33].

In addition to increased drug sensitivity, the *L. pneumophila* Lens TolC defective mutant was highly attenuated for virulence in amoebae and macrophages. Actually, the TolC defective mutant was unable to multiply within host cells from the early steps of host invasion. It exhibits the same defect of virulence as that which was described for the model avirulent *dot*A mutant. The DotA protein belongs to the Dot/Icm type IVB secretion system which is required for replication in amoebae and macrophages and today is thought to secrete more than one hundred substrates [34], [35]. This result demonstrates for the first time the key role of the TolC protein in virulence of *L. pneumophila*. The attenuated virulence phenotype associated with TolC absence has recently been reported in other Gram-negative bacteria [12], [14], [36], [37].

In conclusion, we have demonstrated the major role of the TolC protein in *L. pneumophila* MDR as a component in efflux pump systems. Moreover, TolC has to be considered as a new virulence factor in *L. pneumophila* by its essential role during the early steps of invasion of both amoebae and macrophages. This role could be due to TolC involvement in type I secretion mechanisms. Further work will focus on the determination of TolC partners and substrates of TolC-dependent systems in *L. pneumophila*. These findings may contribute to understanding the molecular mechanisms involved in the export of molecules in *L. pneumophila*. and represent a step towards the development of novel therapeutic agents, especially for affecting efflux or secretion systems [38].

## Materials and Methods

### Bacterial Strains, Plasmids, Media and Growth Conditions

Bacterial strains and plasmids used in this study are summarized in Table S1. *L. pneumophila* serogroup 1 strain Lens (Lp01) was isolated from a patient and is a kind gift of Jérome Etienne and Sophie Jarraud from the CNRL (Centre National de Référence des Légionelles, Lyon, France). *L. pneumophila* strains were grown at 30°C either on buffered charcoal yeast extract (BCYE) agar (Difco) or in BYE liquid medium; each media supplemented with chloramphenicol (Cm; 5 µg/ml), kanamycine (Km; 10 µg/ml) or sucrose (5%) where appropriate. *Escherichia coli* strains were grown at 37°C in LB medium supplemented with chloramphenicol (Cm; 5 µg/ml) or kanamycin (Km; 50 µg/ml).

### Cells Culture

Axenic *Acanthamoeba castellanii* cells were grown on PYG medium (Proteose yeast extract glucose medium) at 30°C and split once or twice a week. *Dyctiostelium discoideum* axenic strain DH1 (ax3; DBS0266325 identification on dictybase http://dictybase.org/) was obtained from François Letourneur (Laboratoire de Transport et Compartimentation Intracellulaire, Institut de Biologie et Chimie des Protéines, UMR 5086 CNRS, IFR 128 BioSciences Lyon-Gerland 7, passage du Vercors, 69367, Lyon, France) and were grown at 25°C in HL5 medium. Macrophage-like U937 cells obtained from Maëlle Molmeret (INSERM E230 Faculté de médecine RTH Laennec 7–11 rue Guillaume Paradin 69372 Lyon Cedex 08) were maintained at 37°C and 5% CO2 in rpmi 1640 tissue culture medium supplemented with 10% heat-inactivated fetal calf serum. Prior to infection, the cells were differentiated in 96 well tissue culture plates for 48 h, using phorbol 12-myristate 13-acetate. Differentiated cells are non replicative, adherent, macrophage-like cells.

### Electroporation of *L. pneumophila* and Screening of Transformants

To prepare competent cells, *L. pneumophila* grown on BCYE agar plates were resuspended in 200 ml of sterile water to an OD600 between 0,5–1. The suspension was divided in four 50 ml tubes (falcon) that were subjected to centrifugation at 4500 rpm during 10 min at 4°C. The bacterial pellet was washed twice with 30 ml of sterile water and the cells were resuspended in glycerol 30%. Competent cells obtained were immediately used or conserved at −80°C as 100 µl samples for long term storage. For electroporation, 3 µl of a plasmid preparation was added to an aliquot of competent cells and submitted to 2,5 kV 600 Ohms and 25 µF using a Biorad electroporation apparatus. Then cells were inoculated in 900 ml BYE liquid medium and incubated at 30°C for 60 minutes before plating on BCYE agar containing the appropriate antibiotic or sucrose for selection.

### Construction of Plasmids

To obtain a *L. pneumophila* Lens mutant defective for *tolC*, a homologous recombination strategy was choosen. A derivative plasmid of pCDP05 was constructed in the laboratory. pCDP05 plasmid is a suicide vector which was used in a previous study to obtain random insertions of a kanamycine resistance cassette on the chromosome of *Legionella pneumophila* [39]. This plasmid bears the *sacB* gene of *Bacillus subtilis* which expression in the presence of sucrose leads to the accumulation of levans at noxious concentrations to Gram-negative bacterial cells when accumulated in periplasmic space. The derivative plasmid was obtained by the deletion of a 4,3 kb *Not*I fragment containing the kanamycine cassette flanked with the two IS10 sequences and *ats1ats2* sequences (alteration of target site recognition). The resulting plasmid, p695, with a unique *Not*I restriction site, confers chloramphenicol resistance and is counter-selectable in presence of sucrose. Two fragments in 5′ and 3′ region of *tolC* were amplified using the primers pairs P1/P2 (amplification of the 5′ region) and primers P3/P4 (amplification of the 3′ region) (Table S2). Primers were flanked with *Not*I restriction site for primers P1 and P4 or *Sal*I restriction site for P2 and P3. The two fragments generated by couple of primers P1/P2 and P3/P4 were digested with SalI enzyme and ligated. PCR was made on the product of ligation using primers P1 and P4. The resulting fragment of approximatively 1 kb was then digested with *Not*I for subcloning in p695. The plasmid p695 with the insertion of P1/P4 fragment was digested with SalI enzyme to insert a kanamycine resistance cassette between the two fragments corresponding to 5′ and 3′ region of *tolC*. The resulted plasmid was named pMF1.

A complementation plasmid was also constructed. The plasmid pML005, derived from PUC18 with Cm cassette in exchange of *bla* gene and a mutation in the ColE1-type replication promoter (Table S1) which was previously shown to confer a stability to the plasmid in *L. pneumophila* [18], was used to clone *tolC* gene under the control of a constitutive promoter (*Pkan*, constitutive promoter of *kan* gene from plasmid pCDP05).

### Construction of *L. pneumophila* Lens *tolC::kan* Strain

The plasmid pMF1 was electroporated in *L. pneumophila*. Kanamycine resistant clones of *Legionella* were plated on BCYE agar containing kanamycine and sucrose for selection of recombinants. The recombinants obtained were controlled by PCR on chromosome, by sequencing and by southern blot analysis. PCR with primers P1/P4 allowed the amplification of a 2 kb fragment which, after *Sal*I restriction, gave two type of fragments: a 1 kb fragment corresponding to the kanamycine cassette and fragments at 500 pb corresponding to the two 5′ and 3′ region of the *tolC* gene. Southern blot on the chromosome digested by BamHI and BglII restriction enzymes was use to reveal the insertion of the kanamycine cassette.

### Multidrug Sensitivity

Sensitivity to different drugs as detergent, dyes, antibiotics and metals was tested by an agar dilution method. Briefly, solution of drug were made in water or ethanol: sodium dodecyl sulfate (SDS; Euromedex), hexadecyltrimethylammonium bromide (CTAB; Sigma-Aldrich), Polymixin B (Sigma-Aldrich), Benzalkonium chlorid (Sigma-Aldrich), Tetracyclin (Sigma-Aldrich), Erythromycin (Sigma-Aldrich), Sodium Déoxycholate (Sigma-Aldrich), Nalidixic acid (Serva), Norfloxacin(Sigma-Aldrich), Ethidium Bromid (Sigma-Aldrich), Acridin Orange (Sigma-Aldrich), Methylene Blue (Prolabo), Rhodamine 6G (Sigma-Aldrich), zinc sulfate heptahydrate (Prolabo), nickel sulfate (Sigma-Aldrich), manganese sulfate (Sigma-Aldrich), Cobalt (Co(II) chloride hexahydrate) (Acros organics). Test drugs were then diluted by twofold in sterile water in a 24 multiwell plate to obtain a final volume of 500 µl of each dilution. Then 500 µl of BCYE agar in surfusion (60°C) was added to each drug dilution and immediately mixed.

The day of the test, *L. pneumophila* cells from fresh BCYE agar plates (4 days of growth at 30°C) were resuspended in sterile water and adjusted to a final suspension with an OD_600nm_ of 5. 10 µl of the bacterial suspension was added into each well of the plate with different dilutions of the drugs. After 5 days at 30°C, the growth into each dilution well was visualized. The well corresponding to the lowest concentration of the drug with no visible sign of growth was reported as MIC 100 for Minimal Inhibitory Concentration of the drug leading to 100% of lethality of bacterial cells.

### Ethidium Bromide Accumulation Assay

This method was based on the method already described to study active efflux in *Salmonella enterica* strains [40]. Bacteria were grown in liquid medium (BYE) at 30°C until stationnary phase corresponding to an optical density of 3.6–3.9. The cells where centrifuged for 5 min at 13 000 rpm and the pellet was washed twice with sterile water. The OD_600nm_ of the final cellular supension was adjusted to 0.3 in sterile water and the cells were incubated or not with carbonyl cyanide m-chlorophenylhydrazone (CCCP; 2,5 mg/L) for 30 minutes. After incubation, ethidium bromide was added to the suspension at a final concentration of 0,005 mg/mL and the bacterial suspension was distributed by aliquots of 100 µL in a 96 multiplate well. The change in fluorescence was recorded every 2 minutes on a « Xenius » (Safas) spectrofluorimeter (excitation 518 nm; emission 605 nm).

### Measurement of Bacterial Release and Intracellular Growth

Intracellular growth of *L. pneumophila* strains was assayed using three eukarytotic hosts: two protozoan cells *A. castellanii* and *D. discoideum* and one mammalian cell: U937 macrophages. These hosts were choosed for there implication in environmental spreading of *L. pneumophila* (protozoa, natural host) or for there role in a clinical infection (macrophages, defective host). *L. pneumophila* were grown on BCYE agar for five days at 30°C prior to infection of protozoan cells and three days at 37°C before the infection of U937 macrophages. *A. castellanii* cells, *D. discoideum* cells and U937 macrophages were first seeded in plates of 96 multiwell plate to a final concentration of 1×10^5^ cells.ml^−1^ in PY, MB and RPMI medium respectively. After a two-hour period of adhesion (except for U937 macrophages with a 2 day differenciation prior to infection) cells were washed four times and *L. pneumophila* was added to an MOI of 10 (in triplicate). The plates were spun at 2000×g for 10 min followed by an incubation of 1 hour (30°C for *A. castellanii*, 37°C for macrophages and 25°C for *D. discoideum*). At the end of this infection period, monolayers were washed four times with tissue culture medium to remove non-adherent bacteria. The time point at the end of the final wash was the initial time point (To). After several times post-infection (0, 12, 24, 48 and 72 h) aliquots of the supernatant were diluted on BCYE agar plates for enumeration of extracellular bacteria. For enumeration of intracellular bacteria the monolayers were washed at different times post-infection and were disrupted either hypotonically (serile water for U937 cells) or with a mild detergent (0,04% Triton X100 for *A. castellanii* and *D. discoideum*). Bacteria were then diluted in sterile water and plated on BCYE agar for enumeration. We verified that our mutant (strain MF201) exhibited the same sensitivity to detergent at the concentration we used. The experiment was repeated at least twice for each infection.

### Cytotoxicity to U937 Cells, *A. castellanii* and *D. discoideum*

For measurement of the number of viable cells remaining, the monolayers were treated with 10% Alamar blue (Invitrogen) as recommended by the manufacturer. Briefly, at the time point indicated monolayers were washed four times with the appropriate medium and then 100 µl of the medium containing 10% (v/v) of Alamar blue was added in each well. After an incubation of several hours (4 h for macrophages, 9 h for *A. castellanii* and approximatively 48 h for *D. discoideum*), measurements of the optical density were performed at a wavelength of 570 nm and corrected for background at 600 nm with a µquant microplate reader. The relative degree of macrophage or amoeba cytotoxicity was expressed as the ratio of the optical density value of an infected monolayer to that of uninfected one with the formula {1-(mean OD value of infected/mean OD value of uninfected} x 100%.

### Study of Invasion of U937 Cells, *A. castellanii* and *D. discoideum*

The study of levels of invasion were performed using a gentamycin protection assay. Briefly after two times of incubation of bacteria with eukaryotic cells (30 and 60 min), monolayers were washed and treated with gentamycin (50 µg/ml) for 1 hour. Then, monolayers were washed to remove gentamycin and were disrupted (either hypotonically for U937 macrophages or with Triton 0,04% for protozoa) to collect intracellular bacteria. Bacteria were diluted in sterile water and plated on BCYE agar for colony enumeration.

### Statistical Analysis

All the results of statitical analysis were obtained using a student's t-test. All the t-test results mentioned correspond to the comparison with the parental strain value in the same conditions. R software (http://www.R-project.org) was used.

## Acknowledgments

We thank Maëlle Molmeret and Christophe Ginevra (INSERM E230 - Faculté de médecine RTH Laennec 7–11 rue Guillaume Paradin 69372 Lyon Cedex 08) for the help with the macrophages infection experiments.

## Supporting Information

Figure S1 Growth kinetics of *L. pneumophila* Lens derivatives. Strains lp01 (WT), MF201 (*tolC::kan*), MF213 (pML005-*tolC*), MF214 (pML005) and lpl701 (*dotA::kan*) were inoculated to an OD600 = 0.2 into BYE medium and grown at 30°C. OD600 at the time points were read using a microplate reader. The results are the mean of two experiments performed in triplicate. Error bars represent standard deviation.
(0.72 MB RTF)

Table S1 Bacterial strains and plasmids
(0.02 MB RTF)

Table S2 List of primers used in this study
(0.02 MB RTF)
