# Supplementary material for: The TolC Protein of Legionella pneumophila Plays a Major Role in Multi-Drug Resistance and the Early Steps of Host Invasion
Source: PLoS One. 2009 Nov 4;4(11):e7732. doi: 10.1371/journal.pone.0007732 (PMC2766832; doi:10.1371/journal.pone.0007732)
Supplement: Table S1 — Bacterial strains and plasmids (0.02 MB RTF) [file pone.0007732.s002.rtf]

Strain or plasmid	Relevant properties	Reference	

E.coli
DH5 

13106
13166

L.pneumophila
Lp
MF201
MF213
MF214
LPL701

Plasmids
pCDPO5

p695 
pMF1

pUC18cm
pML005 

pML005-tolC

	

fhuA2 Δ(argF-lacZ)U169 phoA glnV44 Φ80 Δ(lacZ)M15 gyrA96 recA1 relA1 endA1 thi-1 hsdR17
DH5/pMF1
DH5/pK736


L. pneumophila Lens wild type strain
Lp01 tolC::Kan
MF201/pML005-tolC
MF201/pML005
dotA ::Kan


sacB, ats1ats2, OriT OriV, cm, kan (kanamycin cassette flanked with IS10 sequence)
sacB, OriT OriV, cm (pCPO5 with a deletion of a 4,3 kb fragment)
sacB, cm, kan (p695 with the insertion of a kanamycine cassette between 5'and 3' region of L. pneumophila Lens tolC)
pUC18 with Cm cassette in exchange of bla gene
cm (pUC18cm with the deletion of BstUI site at position 1476 conferring stability without selection in Legionella)
L. pneumophila Lens tolC under kanamycin promotor control in pML005 (insertion between BamH1/SalI restriction sites)	

Taylor et al. (a)

This study
This study


CNRL, Lyon 
This study
This study
This study
This study


Pope et al. (b)

This study
This study

This study
This study

This study

	

a. Taylor RG, Walker DC McInnes RR (1993) E. coli host strains significantly affect the quality of small scale plasmid DNA preparations used for sequencing. Nucleic Acids Res 21: 1677-8
b. Pope CD, Dhand L, Cianciotto NP (1994) Random mutagenesis of Legionella pneumophila with mini-Tn10. Fems Microbiology Letters 124: 107-111.
