# Supplementary material for: The TolC Protein of Legionella pneumophila Plays a Major Role in Multi-Drug Resistance and the Early Steps of Host Invasion
Source: PLoS One. 2009 Nov 4;4(11):e7732. doi: 10.1371/journal.pone.0007732 (PMC2766832; doi:10.1371/journal.pone.0007732)
Supplement: Table S2 — List of primers used in this study (0.02 MB RTF) [file pone.0007732.s003.rtf]

Primer	séquence (5'-3')*	  position	
P1 	CAAATTTTACGCGGCCGCAAGTTGTTTGCG	  -161 -131 (upstream)	
P2	CTTTGGCCTGGTCGACTTTGGC	    514-537  (internal)	
P3	ATTTTCAGTCGACGAGTGAAAAACT	    1180-1204 (internal)	
P4	GAATGAATCTTTGCGGCCGCACAGACATAC	   +116 +148 (downstream)	

* NotI and SalI sites are in bold italic
