# Supplementary material for: The TolC Protein of Legionella pneumophila Plays a Major Role in Multi-Drug Resistance and the Early Steps of Host Invasion
Source: PLoS One. 2009 Nov 4;4(11):e7732. doi: 10.1371/journal.pone.0007732 (PMC2766832; doi:10.1371/journal.pone.0007732)
Supplement: Supplementary file 3 [file pone.0007732.s001.rtf]

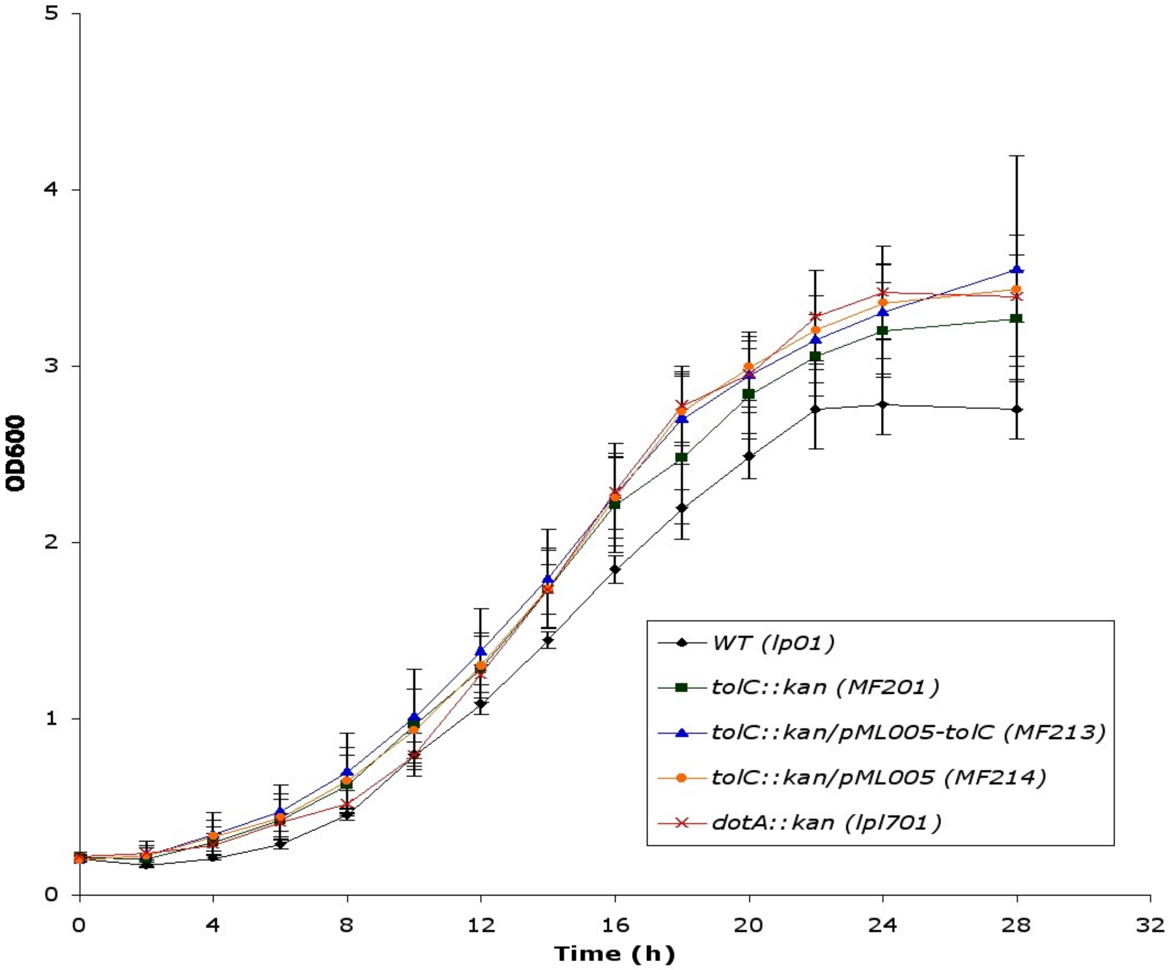

OD600
Time (h)
0
1
2
3
4
5
0
4
8
12
16
20
24
28
32
WT (lp01)
tolC::kan (MF201)
tolC::kan/pML005-tolC (MF213)
tolC::kan/pML005 (MF214)
dotA::kan (lpl701)
